# Comment on Mercurio et al. Outcomes of Acute Achilles Tendon Repair with Platelet-Rich Plasma Augmentation: A Comprehensive Review of Comparative Studies. *J. Clin. Med.* 2026, *15*, 4148

**DOI:** 10.3390/jcm15197530

**Published:** 2026-09-28

**Authors:** Filippo Spiezia, Francesco Oliva, Nicola Maffulli

**Affiliations:** 1 Department of Health Sciences, University of Basilicata, 85100 Potenza, Italy; 2 Department of Orthopaedics and Trauma Surgery, San Carlo Hospital, 85100 Basilicata, Italy; 3 Department of Orthopaedics and Trauma Surgery, Università degli Studi di Roma San Raffaele Telematica, 00166 Rome, Italy; 4 Department of Trauma and Orthopaedic Surgery, University La Sapienza, 00185 Rome, Italy; 5 Centre for Sports and Exercise Medicine, Barts and the London School of Medicine and Dentistry, Mile End Hospital, Queen Mary University of London, London E1 4DG, UK

We read with interest the comprehensive review by Mercurio et al. on platelet-rich plasma (PRP) augmentation in acute Achilles tendon repair [1]. The authors are commended for synthesising comparative evidence on this clinically important topic. However, several methodological concerns limit the interpretability of their conclusions.

The review incorporates only seven comparative studies, enrolling a total of 207 patients, with marked heterogeneity in PRP preparation protocols, outcome measures, and follow-up durations. Although the authors acknowledge this heterogeneity, the conclusion that PRP “may enhance early recovery” demands caution in the light of limited sample sizes and inconsistent findings across studies. Of the three randomised controlled trials (RCTs) included in the review, two—Yasui et al. and Schepull et al.—reported no long-term functional benefit from PRP augmentation after surgical repair [2,3]. The third RCT, by Zou et al., reported statistically significant improvements in long-term ankle range of motion in the PRP group compared with controls [4]. The randomised evidence is therefore heterogeneous rather than uniformly negative, a nuance that should be more explicitly reflected in the review’s conclusions.

A further concern is that the review may overinterpret early functional improvements reported in non-randomised studies. Sánchez et al. described faster recovery of range of motion and earlier resumption of sporting activity in a small case–control series of 12 athletes [5]; however, this finding has not been consistently replicated. For example, the prospective comparative (non-randomised) study by De Carli et al., which is cited by the review, enrolled 30 patients undergoing a combined mini-open and percutaneous repair technique, assigning them alternately to PRP infiltration at surgery and at 14 days postoperatively (Group B) or to a control group (Group A), and found that PRP conferred no superior clinical or functional results [6]. Visual analogue scale (VAS) pain scores, Foot and Ankle Outcome Score (FAOS), and the Victorian Institute of Sport Assessment-Achilles (VISA-A) scale showed no statistically significant differences between groups at 1, 3, 6, or 24 months; isokinetic strength testing and jumping evaluations likewise revealed no benefit. These null findings directly challenge the “early recovery” claim and are not granted sufficient discussion in the review.

The review also omits several studies that provide important additional context to its central conclusions. The two-year follow-up of the PATH-2 randomised, placebo-controlled superiority trial (*n* = 230), the largest single RCT of PRP for acute Achilles tendon rupture, confirmed the absence of clinically meaningful benefit, with similar Achilles Tendon Total Rupture Score (ATRS) and no re-ruptures between 24 weeks and two years [7]. A systematic review and meta-analysis by Boksh et al., which pooled data from six studies (*n* = 510 patients), found similar heel endurance, isokinetic strength, calf circumference, range of motion, and patient-reported outcomes between PRP-treated and control groups, with no significant difference in the re-rupture rate (OR 1.13, 95% CI 0.46–2.80; *p* = 0.79) [8]. Furthermore, a 2025 systematic review and meta-analysis by Yang et al., which is the largest to date with 33 studies and 35,896 patients, concluded that PRP did not yield superior clinical or functional outcomes regardless of whether surgical or conservative treatment was implemented [9]. This incomplete literature coverage limits the interpretive context available to readers, although the omission alone does not in itself establish selective or biassed reporting by the authors.

In summary, the current body of evidence remains inconclusive regarding a durable clinical benefit of PRP as an adjunct to acute Achilles tendon repair, reflecting substantial heterogeneity in PRP protocols and a paucity of large, homogeneous, high-quality randomised trials, rather than definitive proof of ineffectiveness. We respectfully suggest that the authors moderate their conclusions to more explicitly reflect the heterogeneity of the randomised evidence; distinguish clearly between narrative reviews, systematic reviews, and meta-analyses when discussing the wider literature; and discuss the additional randomised and meta-analytic evidence outlined above. Given the marked heterogeneity in PRP preparation, surgical technique, and outcome measures across studies, a qualitative narrative synthesis, as adopted by the authors, represents a reasonable methodological choice at present; nonetheless, a future systematic review and meta-analysis, undertaken once a sufficient number of methodologically comparable randomised trials become available and adhering to PRISMA 2020 guidelines, would help clarify the true clinical value of this intervention.

## Data Availability

No new data were created or analyzed in this study. Data sharing is not applicable to this article.
